# Nucleophosmin1 Is a Negative Regulator of the Small GTPase Rac1

**DOI:** 10.1371/journal.pone.0068477

**Published:** 2013-07-16

**Authors:** Younes Zoughlami, Anne M. van Stalborgh, Paula B. van Hennik, Peter L. Hordijk

**Affiliations:** 1 Department of Molecular Cell Biology, Sanquin Research and Landsteiner Laboratory, Academic Medical Center, University of Amsterdam, Amsterdam, The Netherlands; Children’s Hospital Boston, United States of America

## Abstract

The Rac1 GTPase is a critical regulator of cytoskeletal dynamics and controls many biological processes, such as cell migration, cell-cell contacts, cellular growth and cell division. These complex processes are controlled by Rac1 signaling through effector proteins. We have previously identified several effector proteins of Rac1 that also act as Rac1 regulatory proteins, including caveolin-1 and PACSIN2. Here, we report that Rac1 interacts through its C-terminus with nucleophosmin1 (NPM1), a multifunctional nucleo-cytoplasmic shuttling protein with oncogenic properties. We show that Rac1 controls NPM1 subcellular localization. In cells expressing active Rac1, NPM1 translocates from the nucleus to the cytoplasm. In addition, Rac1 regulates the localization of the phosphorylated pool of NPM1 as this pool translocated from the nucleus to the cytosol in cells expressing activated Rac1. Conversely, we found that expression of NPM1 limits Rac1 GTP loading and cell spreading. In conclusion, this study identifies NPM1 as a novel, negative regulator of Rac1.

## Introduction

Cell migration is vital for many physiological activities, such as organogenesis, wound healing and immune responses. Cell motility is controlled by the actin cytoskeleton, which determines cell polarity, contributes to the formation of adhesive structures and, most importantly, drives forward movement by inducing both protrusive forces at the front and contraction at the lateral sides and rear of the cell [1]. In addition to the actin cytoskeleton, the microtubule network also contributes importantly to cell migration. For example, vesicular transport along the microtubule filaments allows specific spatio-temporal localization of important signaling proteins. This step is important for inducing and maintaining cell polarity which, in turn, is essential for persistent, directional movement of the cell [2], [3].

Cytoskeletal dynamics and cellular adhesion are regulated through signaling by Rho-like small GTPases, such as RhoA which controls myosin-based contraction, and CDC42 and Rac1, that induce actin polymerization and membrane protrusions at the leading edge [1]. These GTPases act as molecular switches, cycling between an inactive GDP-bound state and an active GTP-bound state. This cycling is regulated by guanine nucleotide exchange factors (GEFs) that promote the exchange of GDP for GTP [4] and by GTPase-activating proteins (GAPs) that stimulate the intrinsic GTPase activity [5]. Rho GTPase activity is also regulated by Rho guanine nucleotide dissociation inhibitor (RhoGDI), which binds to inactive RhoGTPases in the cytosol and controls the cytosol-to-membrane translocation of the GTPase [6]. This is key to specific Rho GTPase function, since most GTPases require membrane localization for proper activation and subsequent downstream signaling.

One of the most studied Rho GTPase is Rac1 [7]. Rac1 contributes to cell proliferation, participates in the signaling pathway promoting cell survival and is known for its central role in the control of cell adhesion and cell migration. Following activation, Rac1 interacts with a series of downstream targets, such as p21-activated kinase1 (Pak1) that regulates cytoskeletal dynamics and cell adhesion [8]. Importantly, Rac1-mediated actin polymerization and consequent membrane ruffling at the leading edge are regulated through the WAVE/Arp2/3 complex which controls actin polymerization and branching [9].

The members of Rho family GTPases show high sequence homology. The functional difference between the various family members is explained by their different localization in cells and their binding to different subsets of effector proteins [10]–[12]. Rho GTPase specificity is mainly determined by the hypervariable C-terminal domain. Our laboratory has previously identified a number of proteins that bind to the C-terminus of Rac1 and translocate to cell adhesion sites or the plasma membrane upon Rac1 activation. For example, the adapter proteins caveolin-1 and PACSIN2 are recruited to integrin-regulated focal adhesions and specific tubular structures, respectively, upon Rac1 activation [13]–[15]. Reciprocally, we found that these proteins negatively regulate Rac1 activity. We found that caveolin-1 mediates Rac1 poly-ubiquitylation and degradation and that PACSIN2 targets Rac1 to an endocytic pathway involving GAP proteins.

In this study, we describe the identification of nucleophosmin1 (NPM1) as a novel Rac1 binding protein, which, like caveolin-1 and PACSIN2, acts as a negative regulator of Rac1. NPM1, also known as B23 [16], [17], is a highly conserved, ubiquitously expressed phosphoprotein that shuttles rapidly between the nucleus and cytoplasm [18], although its main location is in the nucleolus. NPM1 is a multifunctional protein regulating various cellular processes, such as ribosome biogenesis, the maintenance of genomic stability and the inhibition of pro-apoptotic pathways [19]–[21]. Nucleo-cytoplasmic shuttling and proper subcellular localization of NPM1 are important determinants for NPM1 function and cellular homeostasis. NPM1 mutations are frequent in acute myeloid leukemia (AML) and are characterized by aberrant NPM1 accumulation in the cytoplasm [19], [22], [23]. Many phosphorylation sites have been identified in NPM1 and different phosphorylation sites have been associated with different functions [20]. NPM1 is phosphorylated by several kinases, including casein kinase 2 and cyclin-dependent kinases [24]–[26].

Here, we show that NPM1 interacts with the C-terminus of Rac1 and negatively regulates Rac1 activity and cell spreading. Importantly, we show that Rac1 activity, in turn, promotes NPM1 nuclear export and alters the NPM1 phosphorylation pattern inside the nucleus. These findings identify a new, bidirectional signaling unit involving two proto-oncogenes NPM1 and the RhoGTPase Rac1.

## Materials and Methods

### Cell Lines and Cell Culture

The Jurkat T-lymphocyte cell line (from the ATCC, Rockville, MO, USA) and HeLa cells were maintained at 37°C and 5% CO_2_ in Iscove’s Modified Dulbecco’s Medium (IMDM; Lonza, Basel, Switzerland) containing 2 mM L-glutamine, 100 U/ml penicillin, 100 µg/ml streptomycin (all purchased from PAA laboratories, Pasching, Austria) and 10% v/v FCS (Bodinco, Alkmaar, The Netherlands. Hela cells were passaged using trypsine (PAA laboratories). HeLa cells were transiently transfected using TransIt (Mirus, Madison, WI, USA) according to the manufacturer’s recommendations.

### Antibodies

The following antibodies were used: anti-GFP (JL8, Clontech), anti-Rac1 (Transduction laboratories), anti-NPM1 (Cell Signaling), anti-phospho NPM1 (recognizes threonine 199, Cell Signaling), rabbit anti-myc and mouse anti-HA (both from Sigma-Aldrich), goat anti-rabbit IgG Alexa 488 or 568 and rabbit anti-mouse IgG Alexa 568 (Invitrogen), Alexa-Fluor-633-labeled Phalloidin (Invitrogen).

### Biotinylated Peptides and GST Fusion Proteins

**Figure 1:**
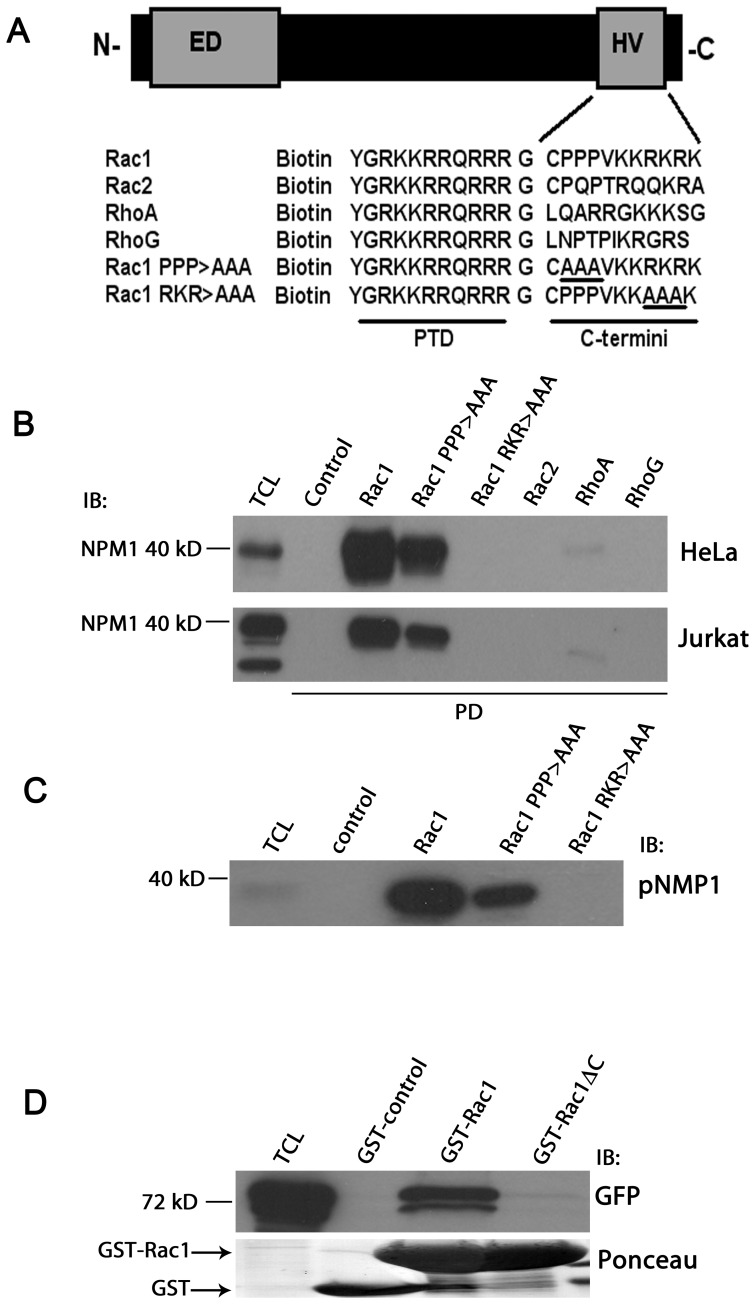
Rac1 interacts through its C-terminus with NPM1. (A) Schematic representation of the Rho-like GTPase C-terminal peptides fused to a protein transduction domain as used in this study. (B) Pull-down (PD) experiments were performed using lysates from HeLa cells (upper panel) or Jurkat T-cells (lower panel) with a control peptide, wild-type and mutant Rac1 C-terminal peptides, Rac2, RhoA and RhoG C-terminal peptides. Association of endogenous NPM1 was detected by immunoblotting (IB) with an NPM1 specific monoclonal antibody (representative example out of three independent experiments is shown). (C) Pull-down (PD) experiment was performed using lysates from HeLa cells with a control peptide, wild-type and mutant Rac1 C-terminal peptides. Association of phosphorylated NPM1 (pNPM1) was detected by immunoblotting (IB) with a phospho-specific NPM1 antibody. (representative example out of two independent experiments is shown). (D) Pull-down (PD) experiment using full-length Rac1 and Rac1 lacking the C-terminus (ΔC) both fused to GST or GST alone was performed with lysates from HeLa cells exogenously expressing GFP-NPM1. Association of NPM1 was detected by immunoblotting (IB) with a GFP specific monoclonal antibody. The ponceau staining shows the presence of the different GST constructs. ED: effector domain of Rac1, HV: hypervariable domain of Rac1, PTD: protein transduction domain, Rac1 PPP→AAA, Rac1 RKR→AAA: Rac1 C-terminal peptide mutants where the three prolines, or RKR sequence were replaced by alanine residues, respectively, TCL: total cell lysates, PD: pull-down, IB: immunoblotting.

Cell permeable peptides containing the amino acid sequence of the C-terminal region of Rac1, the Rac1 C-terminal mutants of the proline motif or polybasic region and the C-terminal region of the related GTPases RhoA and RhoG (Figure 1A) were fused to a protein transduction domain (PTD). These and a control peptide that only comprises the PTD (control) were produced by F-moc protein synthesis (Department of Peptide Synthesis, Netherlands Cancer Institute, Amsterdam, The Netherlands). GST-Rac1 WT and GST-Rac1 lacking the C-terminal tail (ΔC) proteins were produced as described previously [27].

### Pull-down Assays

HeLa cells transiently transfected with the indicated constructs (24 hours) or Jurkat T-cells were washed twice with ice-cold phosphate-buffered saline (PBS; Fresenius Kabi’s Hertogenbosch, The Netherlands) supplemented with 1 mM CaCl2 and 0.5 mM MgCl2 and lysed in NP-40 lysis buffer (i.e. 50 mM Tris-HCl pH 7.5, 100 mM NaCl, 10 mM MgCl2, 10% v/v glycerol and 1% v/v NP-40) to which protease inhibitors (Complete mini EDTA, Roche, Mannheim, Germany) were added. Next, the lysate was centrifuged at 20,000 g for 10 minutes at 4°C. The supernatant was then pre-cleared by incubation with streptavidin-coated beads (Sigma-Aldrich) for 1 hour at 4°C. The pre-cleared lysate was subsequently incubated with the RhoGTPase C-terminal peptide (6 µg) or the control peptide (6 µg) in the presence of streptavidin-coated beads at 4°C for 1 hour with rotation. To determine Rac1 activity, the same procedure was followed and GTP-bound Rac1 was isolated with a biotinylated Pak1-CRIB peptide (20 µg). GST-fusion proteins were coupled to glutathione-coated beads and used at a concentration of 50 µg in each pull-down. GST fusion constructs were incubated with cell lysates at 4°C for 1 hour with rotation.

Cell lysates and pull-down samples were analyzed by SDS-PAGE and immunoblotting using specific monoclonal antibodies and HRP-coupled secondary antibodies.

### Electric Resistance Measurements

For Electrical Cell Substrate Impedance Sensing (ECIS)-based cell spreading experiments, golden ECIS electrodes (8W10E; Applied Biophysics) were treated with 10 µM L-cysteine for 15 minutes and subsequently coated with 5 µg/ml fibronectin in 0.9% NaCl for 1 hour at 37°C. Next, HeLa cells, transfected with the indicated constructs, were seeded at a concentration of 100,000 cells per well in a volume of 400 µl IMDM with 10% FCS. Impedance was measured continuously at 45 kHz using ECIS model 9600. The increased impedance, as a measure of cell spreading, was recorded for 1–2 hours. Measurements and graphs representing the normalized resistance were calculated using the ECIS software (Applied Biophysics).

### Confocal Laser Scanning Microscopy and Data Analysis

HeLa cells were seeded on fibronectin-coated glass coverslips and transfected with the indicated constructs for 24 hours. Cells were then fixed with 3.7% v/v formaldehyde (Merck) in PBS for 10 minutes. When required for antibody staining, cells were washed and permeabilized with 0.5% Triton X-100 in PBS for 5 minutes. Coverslips were then incubated with 2% BSA in PBS at room temperature (RT). Immunostaining was performed using the indicated specific antibodies and secondary labeled antibodies successively for 1 hour at RT. Fluorescent imaging was performed using a confocal laser scanning microscope (LSM510/Meta; Carl Zeiss MicroImaging Inc., Jena, Germany). To quantify NPM1 cytoplasmic localization, we measured the GFP-NPM1 fluorescence intensity of the entire cell as well as only in the nucleus. The fluorescence intensity of the NPM1 signaling in the nucleus was subtracted from the total fluorescence of the entire cell (absolute values) from which the percentage of NPM1 in the cytosol could be calculated. The image processing program ImageJ was used for this analysis and fifteen cells in three independent experiments were quantified.

## Results

### NPM1 Interacts with the Hypervariable C-terminus of Rac1

Our laboratory previously established the association and reciprocal regulation of Rac1 and SET/I2PP2A (inhibitor 2 of protein phosphatase 2A) [28], [29], a nuclear proto-oncogene which, as a protein-fusion with Nup214, is associated with myeloid leukemia [30], [31]. SET binds to Rac1 through its hypervariable C-terminus, specifically through the polybasic region within this domain. Similar to SET, NPM1 is a nuclear protein that has been implicated in the onset of leukemia [18], [19] and that can shuttle from the nucleus to the cytoplasm and vice versa [28]. We therefore questioned whether Rac1 would also interact with NPM1.

To asses whether Rac1 and NPM1 associate, we used a biotinylated peptide encoding the C-terminus of Rac1 and included a series of similar peptides derived from related small GTPases as controls (Figure 1A). Binding to endogenous NPM1 was tested by a streptavidin-based pull-down assay using cell lysates of HeLa or Jurkat T-cells. We found that endogenous NPM1 clearly interacts with the peptide encoding the C-terminus of Rac1 (Figure 1B). In contrast, biotinylated C-terminal peptides derived from Rac2 or RhoG did not bind to NPM1, while weak binding was observed to the RhoA C-terminal peptide (Figure 1B).

The Rac1 C-terminus contains a proline-rich domain and a polybasic domain, that are both involved in the binding of specific regulatory proteins [10]. To examine whether these domains are involved in NPM1 binding, we used the same Rac1 peptide, but now with substitutional alanine mutations of the proline-rich- or polybasic region (Figure 1A). Binding of endogenous NPM1 was minimally affected by mutation of the proline-stretch, while mutating the polybasic region completely abolished the association of NPM1 to the Rac1 C-terminus (Figure 1B).

These data show that NPM1 specifically interacts with the C-terminus of Rac1. In addition, in Figure 1C, we show that the Rac1 C-terminal peptide strongly interacts with the phosphorylated form of NPM1. This interaction was also dependent on the polybasic domain of the Rac1 C-terminus (Figure 1C). Finally, to investigate the interaction between NPM1 and full-length Rac1, we performed a pull-down assay with bacterially purified GST or GST-Rac1 proteins or with GST-Rac1ΔC, which encodes full-length Rac1 that lacks the hypervariable C-terminus, using cell lysates of HeLa cells expressing GFP-NPM1. Our results show that GFP-NPM1 interacts with full-length Rac1 protein and that this interaction is completely abolished by deletion of the C-terminus of Rac1 (Figure 1D). Together, these results show that the Rac1 C-terminus, in particular the polybasic domain, is necessary and sufficient for the interaction of Rac1 with NPM1.

### Rac1 Activity Regulates NPM1 Localization

**Figure 2:**
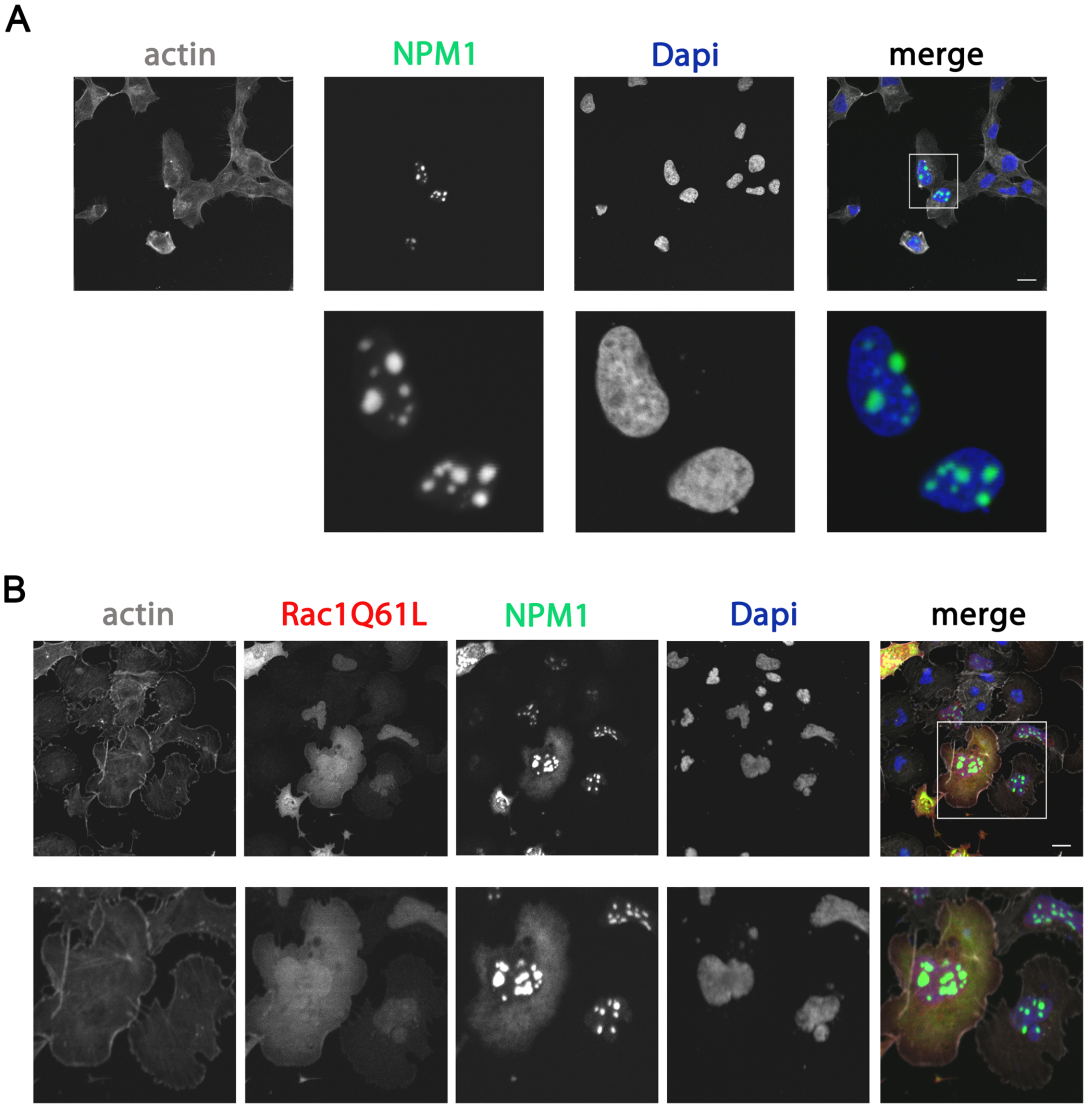
Rac1 activity drives NPM1 out of nucleoli. HeLa cells were grown on glass cover slips and (co)-transfected with GFP-NPM1 (A) or mCherry Rac1Q61L and GFP-NPM1 (B). After 24 hours, cells were fixed and stained with the nuclear dye DAPI and the F-actin binding toxin Phalloidin fluorescently-labeled with Alxa633. Samples were analyzed by confocal laser scanning microscopy. Higher magnification images of the boxed areas are included. Scale bars, 20 µm.

To test if there is also a functional connection between Rac1 and NPM1, we first examined whether Rac1 controls NPM1 localization. We extensively tested different NPM1 antibodies to detect endogenous proteins by immunofluorescence. However, these attempts were without success, which is why we chose to use ectopically expressed proteins for the imaging experiments. We co-transfected HeLa cells with GFP-NPM1 and with a constitutively active Rac1 mutant, Rac1Q61L. As a control for nuclear membrane integrity, we confirmed that expression of the active Rac1 mutant does not alter nuclear histone localization [28]. In cells expressing the GFP-NPM1 construct, NPM1 was mainly detected in nucleoli, identified as small circular structures inside the nucleus (Figure 2A). Interestingly, co-expression of a constitutively active form of Rac1 drove a fraction of GFP-NPM1 out of the nucleus and promoted its accumulation in the cytoplasm (Figure 2B). Quantification of this effect showed that 73±13.8% (mean±sd, n = 15 from three independent experiments) of the total NPM1 pool was located in the cytoplasm in cells expressing active Rac1.

**Figure 3:**
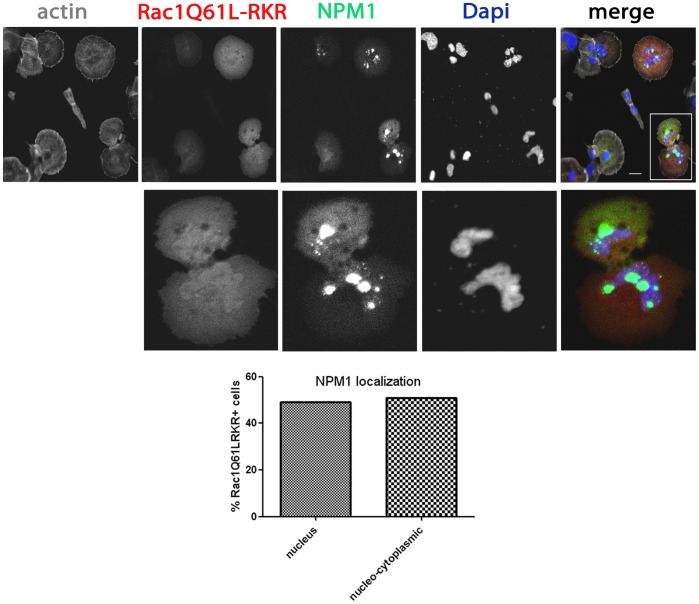
Active Rac1 polybasic mutant has decreased capacity to relocalize NPM1. HeLa cells were grown on glass cover slips and co-transfected with mCherry Rac1Q61L-RKR and GFP-NPM1. After 24 hours, cells were fixed and stained with the nuclear dye DAPI and the F-actin binding toxin Phalloidin fluorescently-labeled with Alexa633. Samples were analyzed by confocal laser scanning microscopy. Higher magnification images of the boxed areas are included. Scale bars, 20 µm. Bar graph represents the percentage of cells (mean±SD) expressing Rac1Q61L-RKR with either NPM1 exclusively in the nucleus or NPM1 in both the nuclear and cytoplasmic compartment (nucleo-cytoplasmic) Fifty cells expressing Rac1Q61L-RKR in two independent experiments were analyzed.

Because the polybasic region of Rac1 mediates its association to NPM1, we anticipated that a constitutively active Rac1 mutant lacking this region would not induce NPM1 relocalization outside the nucleus. Interestingly, in approximately half of the cells expressing the active Rac1 polybasic region mutant, NPM1 showed detectable extra-nuclear localization (Figure 3). This demonstrates that, to a limited extend, the polybasic region is dispensable and that Rac1 activity alone is sufficient to promote NPM1 extranuclear localization.

**Figure 4:**
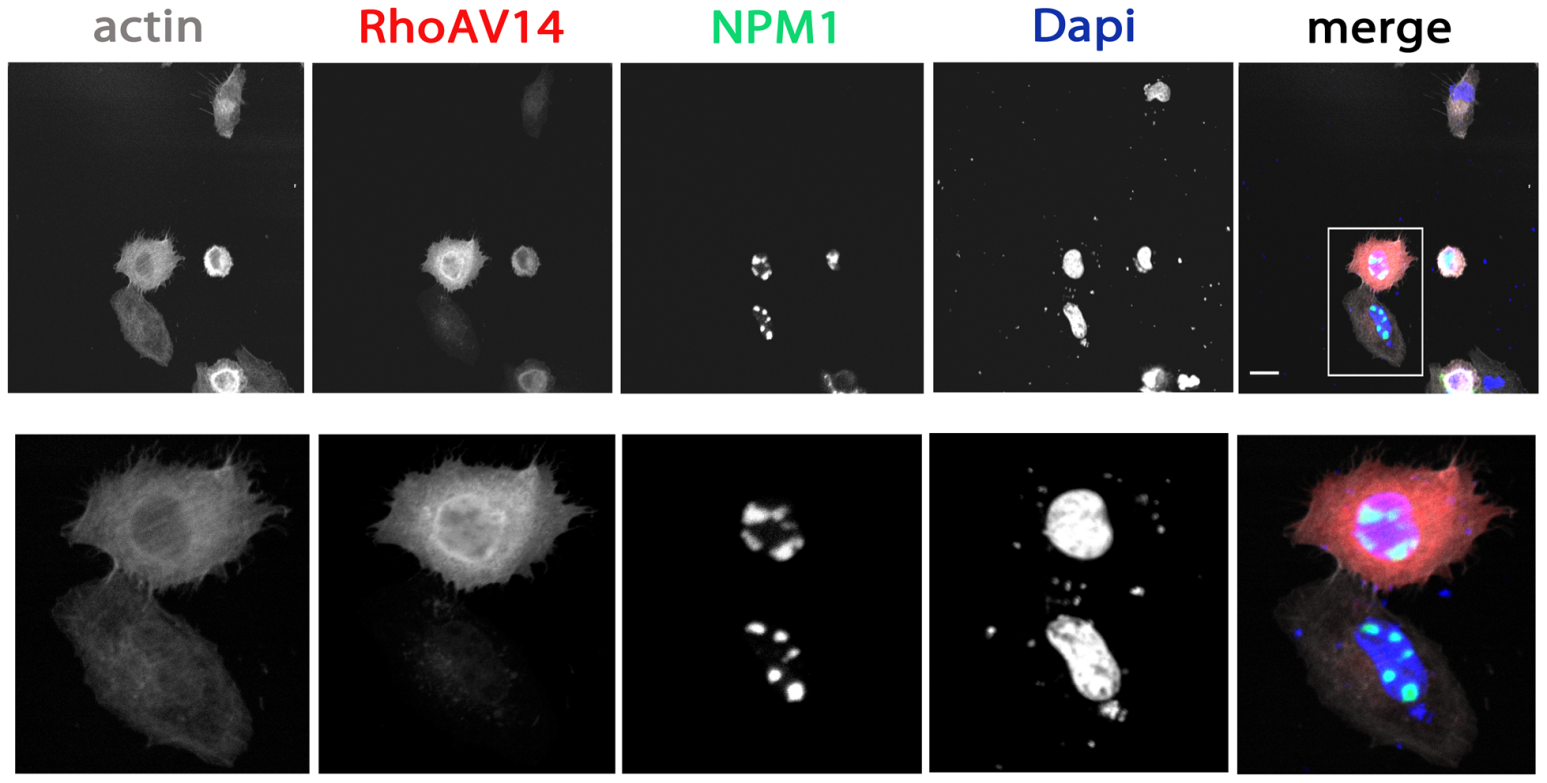
Active RhoA does not affect NPM1 localization. HeLa cells were grown on glass cover slips and co-transfected with HA-tagged active RhoA mutant, RhoAV14, and GFP-NPM1. After 24 hours, cells were fixed and stained with the nuclear dye DAPI and the F-actin binding toxin Phalloidin fluorescently labeled with Alxa633. RhoA was detected by a monoclonal anti-HA antibody followed by an anti-mouse Alexa568 antibody. Samples were analyzed by confocal laser scanning microscopy. Higher magnification images of the boxed areas are included. Scale bars, 20 µm.

We then investigated whether the closely related GTPase RhoA induces the same effects on NPM1 translocation as activated Rac1. Expression of a constitutively active RhoAV14 mutant did not alter NPM1 nucleolar localization (Figure 4), suggesting that Rac1 activity specifically regulates NPM1 subcellular localization.

**Figure 5:**
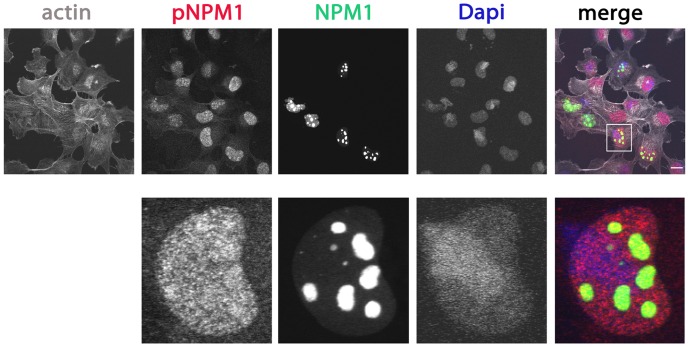
Phospho-NPM1 shows dispersed localization throughout the nucleus. HeLa cells were grown on glass cover slips and transfected with GFP-NPM1. After 24 hours, cells were fixed and stained with a phospho-specific rabbit antibody against NPM1 followed by a goat anti-Rabbit IgG Alexa568, the nuclear dye DAPI and the F-actin binding toxin Phalloidin fluorescently labeled with Alexa633 and analyzed by confocal laser scanning microscopy. Higher magnification images of the boxed areas are included. Scale bars, 20 µm.

Because NPM1 function is regulated by phosphorylation and because the Rac1 C-terminus associates to phosphorylated NPM1 (pNPM1; Figure 1C), we questioned whether Rac1 activity alters the distribution of pNPM1. First, we used a phospho-specific NPM1 antibody to document the localization of pNPM1. This staining revealed that the phosphorylated fraction of endogenous NPM1 localized markedly different from the total NPM1 pool. Phospho-NPM1 was diffusely dispersed throughout the nucleus and also localized to small dotted structures inside the nucleoplasm (Figure 5).

**Figure 6:**
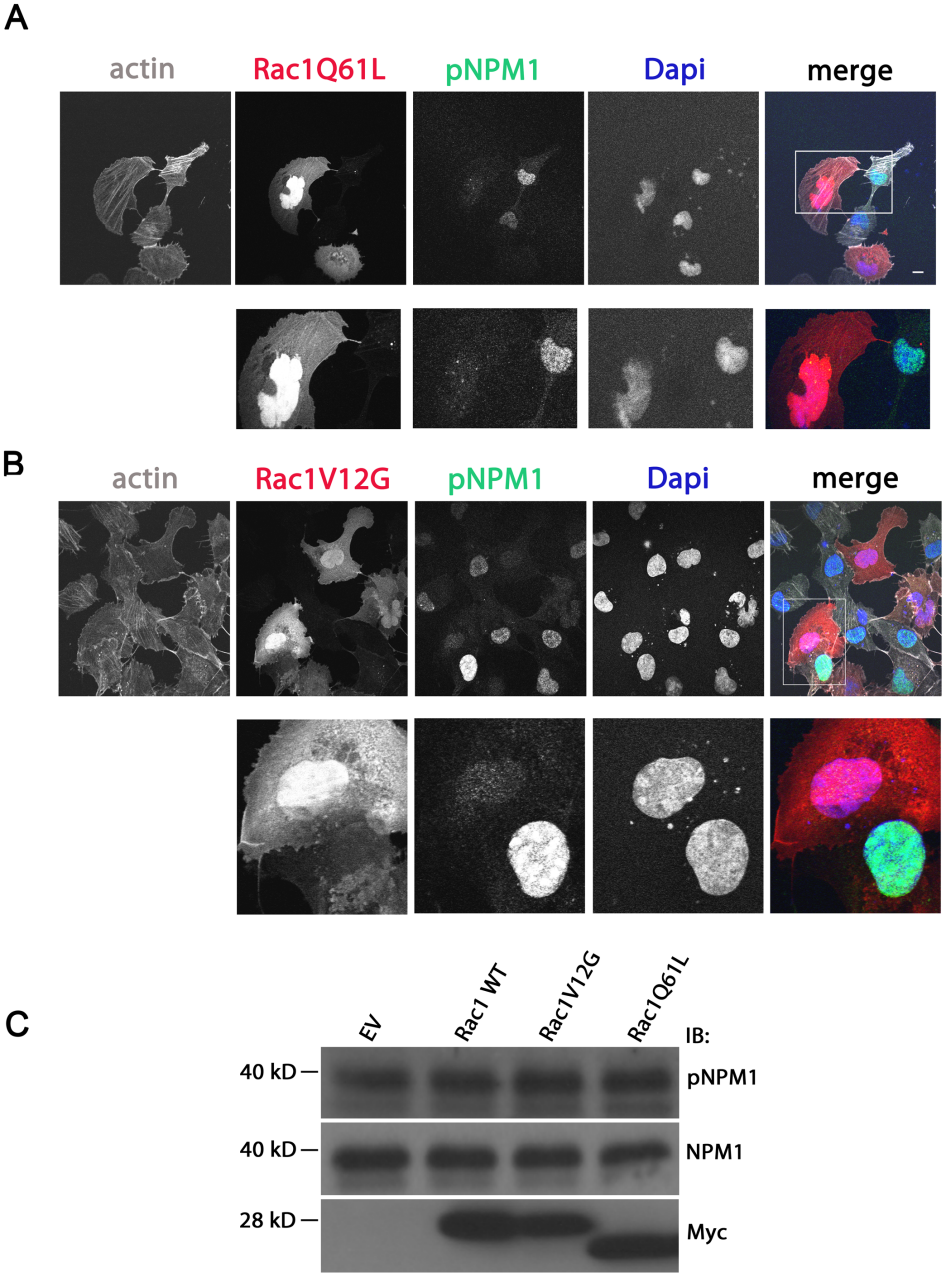
Rac1 activity alters phospho-NPM1 distribution. HeLa cells were grown on glass cover slips and transfected with either mCherry Rac1Q61L (A) or mCherry Rac1V12G (B). After 24 hours, cells were fixed and stained with a phospho-specific rabbit antibody against NPM1 followed by a goat anti-Rabbit IgG Alexa488, the nuclear dye DAPI and the F-actin binding toxin Phalloidin fluorescently labeled with Alexa 633 and analyzed by confocal laser scanning microscopy. Higher magnification images of the boxed areas are included. (C) Hela cells were transfected with an empty vector, myc-tagged Rac1 WT or two different myc-tagged constitutively active Rac1 mutants; Rac1V12G and Rac1Q61L and after 24 hours lysates were made and endogenous NPM1 as well as phospho-NPM1 (pNPM1) were detected by immunoblotting (IB) with a NPM1 specific or a NPM1 phospho-specific antibody. EV: empty vector. Scale bars, 20 µm.

Interestingly, in cells expressing a constitutive active Rac1 mutant, either Rac1Q61L or Rac1V12G, staining of phospho-NPM1 largely disappeared from the nucleus and was no longer clearly detectable (Figure 6A and 6B). This could either be due to a diffuse localization of the pool of pNPM1 (as observed for tot NPM1, Figure 2B) or to Rac1 induced de-phosphorylation of pNPM1. To test for the latter option, we detected pNPM1 levels by Western Blot in lysates from cells expressing either of the active Rac1 mutants. This showed that expression of the Rac1V12G or Rac1Q61L mutants did not affect the levels of endogenous NPM1 nor those of phosphorylated NPM1 (Figure 6C). Collectively, these data show that Rac1 activity induces NMP1 nucleo-cytoplasmic shuttling of NPM1 and induces a diffuse cytoplasmic localization of pNPM1.

### NPM1 Negatively Regulates Rac1 Activity

**Figure 7:**
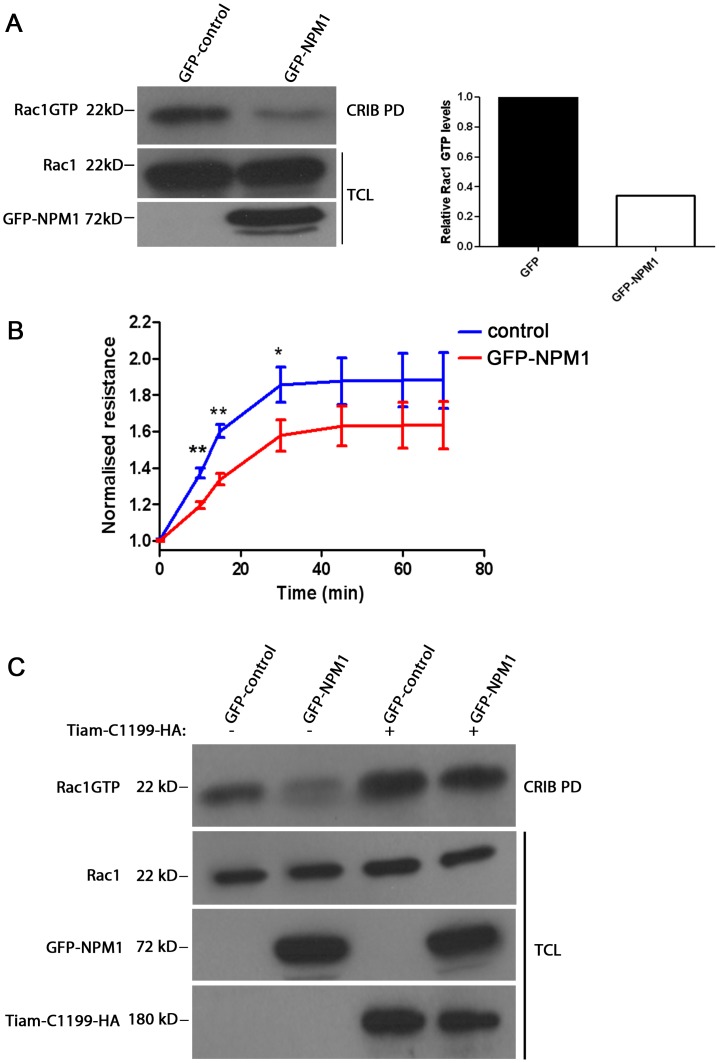
NPM1 is a negative regulator of Rac1. (A) Rac1 GTP loading was measured by biotinylated Pak-CRIB peptide-based pull-down (PD) with lysates of GFP control-transfected HeLa cells or HeLa cells transfected with GFP-NPM1. Rac1, Rac1 GTP and GFP-NPM1 were detected by immunoblotting with Rac1 and GFP specific monoclonal antibodies respectively. The bar graph shows the relative levels of Rac1 GTP levels compared to that in control as determined by quantification of Western blots (representative example out of three independent experiments is shown). TCL: total cell lysates, PD: pull-down. (B) Cell spreading on fibronectin coated ECIS-electrodes was determined for mock-transfected HeLa cells or HeLa cells expressing GFP-tagged NPM1. The results are depicted as normalized mean resistance of three independent experiments. (n = 3) *p<0.05, **p<0.01. (C) Rac1 GTP loading was measured by biotinylated Pak-CRIB peptide-based pull-down (PD) with lysates of GFP control-transfected HeLa cells or HeLa cells transfected with GFP-NPM1 in the absence or presence of the GEF protein Tiam-C-1199 tagged with HA. Rac1 and Rac1 GTP were detected by immunoblotting with Rac1 specific monoclonal antibody. GFP-NPM1 and Tiam-C-1199-HA were detected by immunoblotting with GFP and HA specific monoclonal antibodies, respectively.

Given that NPM1 interacts with Rac1, we set out to determine the functional consequences of this interaction for Rac1 function. We first tested the effect of NPM1 overexpression on the GTP-loading of Rac1. We found that the basal level of endogenous, active Rac1 was clearly decreased in cells transfected with GFP-NPM1 (Figure 7A). Importantly, expression of NPM1 did not affect the total levels of endogenous Rac1, excluding that increased degradation would account for the loss of active Rac1. In line with these data, we found that NPM1 overexpression reduced cell spreading on fibronectin, a process that is dependent on Rac1 activity (Figure 7B). These results suggest that NPM1 acts as a negative regulator of Rac1.

We then questioned whether NPM is able to block GEF-mediated Rac1 activation. To investigate this, we tested whether the Rac1 GEF Tiam1-C1199 is still able to activate endogenous Rac1 in the presence of NPM1. Tiam1-C-1199 is a N-terminally truncated mutant Tiam1 that is widely used because of its enhanced stability and increased activity as compared to full-length Tiam1 [32]. As expected, we observed NPM1-mediated downregulation of Rac1GTP levels in the control situation (Figure 7C). In addition, expression of Tiam1-C1199 increased Rac1GTPlevels. However, co-expression of NPM1 did not reduce Tiam1-C1199-induced activation of endogenous Rac1 (Figure 7C). We also found that NPM1 did not block the activation of Rac1 upon expression of another Rac1GEF, TrioD1 [33] (data not shown). These data show that NPM1 does not interfere with the Rac1 activation by GEF proteins and suggest that NPM1 acts downstream of Rac1 to promote its inactivation, possibly by promoting the association of activated Rac1 to GAP proteins.

## Discussion

The link between Rac1 and NPM1 was previously only described in nucleophosmin-anaplastic lymphoma kinase (NPM-ALK)-positive lymphomas [34], [35]. NPM-ALK is an oncogenic fusion protein, which acts as a constitutive active tyrosine kinase [36]. NPM-ALK signals in part through Rac1 thereby contributing to the pathogenesis of ALK-positive human lymphomas [34], [35]. Here, we present data indicating that NPM1 and Rac1 interact and reciprocally regulate each other.

Like many other regulatory proteins, NPM1 interacts with Rac1 via the hypervariable C-terminus of Rac1. Importantly, the association to NPM1 was specific for Rac1 as compared to other, highly related, small GTPases. This suggested that NPM1 could be a selective regulator of Rac1. Indeed, overexpression of NPM1 reduced GTP loading of Rac1 and cell spreading on fibronectin. We attempted to detect the opposite effect, i.e. increased basal Rac1 activity, upon silencing NPM1 with two different shRNAs. However, NPM1 knock-down did not affect Rac1 GTP loading (data not shown). This may be the result of redundancy as three NPM family members, NPM1, NPM2 and NPM3 have been identified in mammals [20]. Moreover, two isoforms of NPM1 exist [37]. Therefore, it is possible that another NPM homologue or isoform compensates for the loss of the targeted NPM1.

How NPM1 regulates Rac1 activity remains to be established. Rac1 is regulated at, at least, two important levels. The first is at the level of activation, where GEFs play an important role. Proper GEF localization at the plasma membrane, on intracellular vesicles or specific membrane domains, and a cascade of local signaling events are essential for efficient Rac1 activation [4], [38]. However, NPM regulation of the activation of Rac1 appears unlikely, since expression of NPM1 did not interfere with GEF-induced Rac1 GTP loading. The second means of regulating the Rac1 activity cycle is at the level of inactivation. Inactivation is achieved by extracting Rac1 from the plasma membrane followed by GAP-promoted GTP hydrolysis. Some proteins, such as RhoGDI, caveolin-1 and PACSIN2, facilitate Rac1 internalization from the plasma membrane [13], [14], [39], [40]. Most known Rac1-regulatory proteins are localized at the plasma membrane or throughout the cytoplasm. Therefore, it remains unclear how NPM1 regulates Rac1 GTP loading, since NPM1 is localized in nucleoli, which are specialized nuclear compartments. NPM1 is known to rapidly shuttle between the nucleus and the cytoplasm and may limit Rac1 activity during its short stay outside the nucleus. NPM1 contains both a nuclear localization signal (NLS) as well as a nuclear export signal (NES) [21], [41], [42]. However, despite the NES motif, NPM1 is mainly localized in nucleoli [43], indicating that the NLS is dominant. Thus, under physiological conditions, nuclear import of NPM1 outweighs its export. We found that increasing the levels of activated Rac1 in cells reversed this balance and promoted NPM1 recruitment to the cytoplasm, possibly to promote Rac1 inactivation. Finally, Rac1 can be inactivated by the ubiquitin-proteasome system [44], [45], however, this does not appear to play a role here, as expression of NPM did not alter the total levels of the Rac1 protein.

The hypervariable C-terminus of Rac1 comprises a NLS, as a consequence of which a subset of Rac1 resides in the nucleus [46]. A considerable number of effector proteins, scaffolding proteins and GEFs for small GTPases are found in the nucleus [47]. In line with this, various RhoGTPases, including Rac1 and RhoA, are known to regulate gene transcription, proliferation and transformation [48], [49]. A previous study showed that Rac1 translocation to the nucleus was linked to the cell cycle, with nuclear Rac1 promoting, and cytoplasmic Rac1 inhibiting mitosis [50]. Most recently, activating Rac1 mutations were found in various forms of cancer, including melanoma [51]. Activated Rac1 shows, like NPM1, nucleo-cytoplasmic shuttling [52] so it is attractive to suggest that a functional link between Rac1 and NPM1 in controlling gene transcription and proliferation exists. This is also in line with the notion that NPM1 acts as a sensor for oncogenic stress [19]. However, to what extent localization of Rac1 activity to the nucleus and/or nuclear interaction of Rac1 with NPM1 is required for these functions remains to be further investigated. It is intriguing to speculate that NPM1 regulates Rac1 signaling in the nucleus by limiting its activation. We are currently pursuing this issue by the use of NPM1 mutants, exclusively expressed either in the cytoplasm or in the nucleus to clarify the relevant location of NPM1 where it accomplishes its role as a negative regulator of Rac1.

Another key finding of the current study is that Rac1 activity alters the import/export dynamics of NPM1, thereby promoting its translocation into the cytoplasm. Aberrant NPM1 cytoplasmic accumulation is associated with AML, where it is implicated in promoting malignant cell growth [19], [21], [22]. Similarly, our laboratory recently identified the oncogene SET as a target of Rac1 [28], [29]. Constitutive active Rac1 induced the nuclear export of SET into the cytoplasm, which promotes cell migration. Thus, we propose a model in which aberrant Rac1 signaling alters the subcellular localization of several oncogenes and triggers their oncogenic activity. Our finding that the constitutively active Rac1 with a mutated polybasic region relocalizes NPM1 in the cytoplasm in a portion of the transfected cells was unexpected. This mutant is not anticipated to bind NPM1 and also misses the NLS that targets Rac1 to the nucleus. This suggests that active Rac1 is able to initiate a signaling cascade from the cytosol to stimulate NPM1 relocalization. This makes it unlikely that the direct interaction between Rac1 and NPM1 is essential to induce NPM1 translocation from the nucleus. However, in another portion of the transfected cells, transfection with the active Rac1 polybasic region mutant did not affect NPM1 localization in the nucleoli, showing that in addition to Rac1 activity, this domain is required for optimal regulation of NPM1 subcellular localization.

We also found that expression of a constitutively active mutant of Rac1 altered NPM1 phosphorylation pattern. The fraction of phosphorylated NPM1 observed by confocal microscopy became largely undetectable in the presence of an active mutant of Rac1. Since we could not detect significant de-phosphorylation of pNPM1 in the presence of activated Rac1 mutants, this result indicates that Rac1 activity disperses pNPM1 from the nucleus into the cytoplasm, obscuring clear cut detection by microscopy.

In conclusion, we establish NPM1 as a novel Rac1 interactor that acts as a negative regulator of activated Rac1. Like other Rac1-regulatory proteins such as caveolin-1 and PACSIN2, NPM1 and Rac1 showed reciprocal regulation with Rac1 promoting NPM1 nuclear exit. This suggests that NPM1 is part of a negative feedback loop that serves to limit Rac1 signaling. Future experiments are directed at identifying the molecular mechanisms of Rac1 inactivation by NPM1 and to determine the biological consequences of Rac1-induced NPM1 accumulation into the cytoplasm.
